# A scoping review to identify and describe the characteristics of theories, models and frameworks of health research partnerships

**DOI:** 10.1186/s12961-022-00877-4

**Published:** 2022-06-18

**Authors:** B. J. Tittlemier, J. Cooper, D. Steliga, R. L. Woodgate, K. M. Sibley

**Affiliations:** 1 Applied Health Sciences Program, University of Manitoba, 202 Active Living Centre, Winnipeg, MB R3T 2N2 Canada; 2 Department of Occupational Therapy, College of Rehabilitation Sciences, Rady Faculty of Health Sciences, University of Manitoba, R106- 771 McDermot Avenue, Winnipeg, MB R3E 0T6 Canada; 3 Department of Community Health Sciences, Max Rady College of Medicine, University of Manitoba, S113- 750 Bannatyne Avenue, Winnipeg, MB R3E 0W3 Canada; 4 Tier 1 Canadian Institutes of Health Research Canada Research Chair, Rady Faculty of Health Sciences, College of Nursing, University of Manitoba, 89 Curry Place, Winnipeg, MB R3T 2N2 Canada; 5 Department of Community Health Sciences, Max Rady College of Medicine, Rady Faculty of Health Sciences, George and Fay Yee Centre for Healthcare Innovation, University of Manitoba, 753 McDermot Avenue, Winnipeg, MB R3E 0T6 Canada

**Keywords:** Frameworks, Knowledge translation, Models, Research partnerships, Theories

## Abstract

**Background:**

Engaging users of health research, namely knowledge users, as partners in the research process may to lead to evidence that is more relevant to the users. This may optimize the uptake of evidence in healthcare practice, resulting in improved health outcomes or more efficient healthcare systems. However, barriers to involving knowledge users in the research process exist. Theories, models and frameworks may help guide the process of involving knowledge users and address barriers to engaging with knowledge users in research; however, there is little evidence identifying or describing the theories, models and frameworks of health research partnerships.

**Objectives:**

Identify and describe theories, models and frameworks of health research partnerships. Report on concepts of knowledge user engagement represented in identified theories, models and frameworks.

**Methods:**

We conducted a scoping review. Database (MEDLINE, Embase, CINAHL, PCORI) and ancestry and snowball searches were utilized. Included articles were written in English, published between January 2005 and June 2021, specific to health, a research partnership, and referred to a theory, model or framework. No critical appraisal was conducted. We developed a coding framework to extract details related to the publication (e.g. country, year) and theory, model or framework (e.g. intended users, theoretical underpinning, methodology, methods of development, purpose, concepts of knowledge user engagement). One reviewer conducted data extraction. Descriptive statistics and narrative synthesis were utilized to report the results.

**Results:**

We identified 21 874 articles in screening. Thirty-nine models or frameworks were included in data analysis, but no theory. Two models or frameworks (5%) were underpinned by theory. Literature review was the method (*n* = 11, 28%) most frequently used to develop a model or framework. Guiding or managing a partnership was the most frequently reported purpose of the model/framework (*n* = 14, 36%). The most represented concept of knowledge user engagement was principles/values (*n* = 36, 92%).

**Conclusions:**

The models and frameworks identified could be utilized by researchers and knowledge users to inform aspects of a health research partnership, such as guidance or implementation of a partnership. Future research evaluating the quality and applicability of the models and frameworks is necessary to help partners decide which model or framework to implement.

**Supplementary Information:**

The online version contains supplementary material available at 10.1186/s12961-022-00877-4.

## Background

The disconnect between the development of health research and its subsequent utilization in healthcare practice has been well established [1–3]. Underutilization of evidence may impact health and functional outcomes in patients [4, 5], and has been attributed to how evidence has been disseminated with the intended audiences [6]. Additionally, research conducted without the involvement of knowledge users, such as clinicians, patients, caregivers, policy-makers or decision-makers, may contribute to its underutilization because critical components of the research process (e.g. setting priorities, establishing research questions, choosing methods, collecting and analysing data) do not incorporate the perspectives and experiences of the knowledge users. The lack of involvement of knowledge users may result in production of evidence that is irrelevant to them [7, 8]. Research is historically within the purview of academia with responsibility for establishing the research questions and agenda, designing and conducting the study, and disseminating the results [6]. At times, this researcher-driven approach develops evidence that is perceived as irrelevant by knowledge users and results in underutilization of evidence in healthcare practice [7–9].

Approaches to conducting research that involve a partnership between researchers and knowledge users during the research process are now being employed to develop knowledge that is deemed more relevant to knowledge users [6]. These research partnerships are rooted in approaches to evidence development that actively involve knowledge users in any part of the research process [10–12]. Research partnerships aim to develop more meaningful evidence for knowledge users than researcher-driven approaches, thus potentially enhancing implementation and improving health outcomes and the efficiency of a healthcare system or organization [13]. Acknowledging that numerous complementary traditions coexist, such as integrated knowledge translation and community-based participatory research (CBPR), we utilize the term “health research partnerships” and we define it as collaborative research activities specific to health that involve a minimum of (1) one researcher associated with an academic institution and (2) one nonacademic partner such as an organization, clinician, patient, caregiver, policy-maker or decision-maker [7, 10, 12].

Numerous benefits of health research partnerships have been reported in the literature which impact researchers and knowledge users [14–17]. For instance, in an analysis of reviews on research partnerships across all disciplines, Hoekstra et al. [17] reported increased motivation for research projects, more positive attitudes towards research, increased accessibility to healthcare information and enhanced feelings of empowerment, confidence and being valued. Further benefits include increased participant enrolment rates [15, 16], strengthened social networks [14–16] and improved research skills and capacity [15, 17].

The extent of knowledge user involvement may vary within health research partnerships [11, 17], and can be examined using existing criteria, such as the Spectrum of Public Participation developed by the International Association for Public Participation (IAP2) [18]. The IAP2 Spectrum consists of five levels of public participation, namely inform, consult, involve, collaborate and empower, with “inform” representing the lowest level of engagement and “empower” representing the highest (Additional file 1) [18]. The IAP2 Spectrum has been used to classify the level of patient and public participation in selecting and developing patient-reported outcome measures in paediatrics [19].

There have been several calls for research to identify, describe, evaluate and validate theories, models and frameworks (TMFs) of health research partnerships [20–22]. This research is needed to explain why research partnerships succeed or fail, to clarify assumptions about research partnerships, and to help understand at what point and the ways in which to engage with knowledge users [7, 22]. Theories, models and frameworks organize concepts, thinking and observations [23–26]. Furthermore, they offer clarity on various aspects of implementation practice and research, which may explain why they are often grouped together [27]. Models and frameworks are similar in that they are organizational templates that can be used to plan, anticipate challenges, identify performance measures and measure the impact of research partnerships [26, 28]. A theory is a set of connected concepts, definitions and relational statements that present an organized way of observing relationships among variables [24, 25]. A theory can describe, explain and predict a phenomenon [24, 25]. Unlike a model or framework, a theory can explain why a health research partnership was or was not successful or may predict a successful research partnership [22]. Because TMFs can be utilized to deepen our understanding of aspects of health research partnerships, it is necessary to identify, describe, evaluate and validate TMFs of health research partnerships.

Research reviewing and synthesizing TMFs of research partnerships has emerged [7, 29]. Jull et al. [7] sought to identify frameworks of knowledge user engagement, which they defined as “an arrangement in the governance of the research process with those who influence, administer and/or who are active users of healthcare systems and that leads to co-production of knowledge, and associated concepts” (p. 2). Using the Engagement in Health Research Literature Explorer (https://www.pcori.org/engagement/engagement-literature), Jull et al. [7] identified 54 frameworks and 15 concepts (Table 1) of knowledge user engagement that could help researchers and knowledge users operationalize research partnerships. While the concepts identified provide a useful overview of similarities and differences within existing partnership TMFs, Jull et al. [7] did not explicitly identify or describe the characteristics of the identified frameworks, and this research may be needed to evaluate and help select a TMF. Additionally, research to identify and describe TMFs of health research partnerships may advance their use in research and produce more relevant evidence for knowledge users, thus increasing the utilization of evidence in healthcare practice. Therefore, our objectives were threefold: (1) identify TMFs of health research partnerships, (2) describe the characteristics of the identified TMFs of health research partnerships and (3) map each identified TMF to Jull et al.’s [7] 15 concepts of knowledge user engagement.

**Table 1:** Concepts of knowledge user engagement as described by Jull et al. [7]

Concept	Description of collaborative research process
Researcher: prepare, support	Initiate/support researcher capacity/behaviour for power-sharing, expertise, engagement—includes language and knowledge differences, learning (e.g. attending meetings with community groups, volunteering and working with groups to understand knowledge user perspectives)
Knowledge user: prepare, support	Initiate/support knowledge user/community organizational capacity/behaviour for power-sharing, expertise, engagement (e.g. develop resource manual, provide training in research methods)
Relational process	Initiate and/or sustain a relational process (relationship-building) between knowledge user–researcher to promote respect, reciprocity, trust and partnership synergy
Research agenda	Engage in a process to define study agenda: scope, priorities, objective(s)
Ethics: principles/values	Conduct knowledge user–researcher partnership work in an ethical way demonstrated by reflection on ethical concepts and/or concern with particular values, and research conducted in ways reported as meaningful, respectful, inclusive of those in the research partnership. Evidence of principled (versus policy, rules) research conduct
Research questions	Define research questions to identify what, specifically, the research project aims to achieve to justify the need to conduct the research (i.e. how/why was this topic chosen? What gap will it fill?)
Resources	Develop funding applications/grant proposals for and/or to obtain resources (e.g. funding, time) to support knowledge user–researcher engagement
Ethics: policy/rules	Conduct knowledge user–research partnership work in an ethical way demonstrated by participation in an ethical application development (e.g. writing consent forms), review (e.g. research ethics board, community review) and/or development and/or use of an ethical framework (e.g. knowledge user role in the use of particular protocols, processes)
Methodology	Decide on the research methodology (approach) or report process to justify the use of the proposed methodology
Methods	Decide upon research methods and a justification for the use of the proposed methods; selection of outcome measures
Collect data	Collect data and include tool development
Analysis	Decide about the analysis and interpretation of data (e.g. what form of analysis and how it will be conducted)
Disseminate	Identify the appropriate audience to disseminate the research findings and tailor the message and medium to the audience to create tangible products (e.g. publication of findings, community meetings)
Evaluate	Evaluate the research study processes
Sustain	Maintain study benefits at a certain rate, level [i.e. make deliberate efforts to sustain study intervention(s)]

## Methods

Our scoping review followed methodological frameworks outlined by Arksey and O’Malley [30] and Levac et al. [31] The reporting of our scoping review was guided by the Preferred Reporting Items for Systematic Reviews and Meta-Analyses (PRISMA) Extension for Scoping Reviews (PRISMA-ScR) [32]. We developed a scoping review protocol a priori and published it on the Open Science Framework (https://osf.io/qntym) [33]. The steps in our scoping review are discussed below.

### Step 1: establishing the research question(s)

1. What theories, models and frameworks of health research partnerships have been identified and described in the published literature?
2. What are the characteristics of the identified theories, models and frameworks of health research partnerships?
3. What concepts of knowledge user engagement proposed are present in the identified theories, models and frameworks of health research partnerships?

### Step 2: identifying relevant studies

We collaborated with a research librarian to develop our search strategy, which included both controlled vocabulary (e.g. Medical Subject Headings) and free text terms informed by previously published literature (e.g. theory, model, framework, CBPR, participatory action research, patient and public involvement, integrated knowledge translation) [7, 34, 35]. We searched MEDLINE (Ovid), Embase and CINAHL (Cumulative Index to Nursing and Allied Health Literature) for articles from January 2005 to June 2021. The time frame for our search reflects the period of increasing publications specific to research partnerships [7, 35]. Trial searches were conducted from 24 April until 14 May 2020. A final search was conducted on 20 May 2020. We completed an updated search on 23 June 2021. Our full Ovid search strategy can be found in Additional file 2. The Ovid search strategy was adapted and applied to Embase and CINAHL.

We also searched the Engagement in Health Research Literature Explorer because it is an open-access database that consists of peer-reviewed articles related to engagement in health research (https://www.pcori.org/engagement/engagement-literature). This online repository of literature was developed by the Patient-Centred Outcomes Research Institute (PCORI), and the collection of articles in the PCORI Explorer is kept up to date with regular searches of PubMed and MEDLINE [36]. For details on the search terms and search strategy that PCORI staff members utilize to search PubMed and MEDLINE for applicable articles, please see: https://www.pcori.org/engagement/engagement-health-research-literature-explorer/engagement-health-research-literature-explorer-supplemental-methods-information. We searched the PCORI Explorer from January 2018 to June 2021 to capture research that was not previously included in Jull et al. [7]. The articles in PCORI can be searched via article topic type, types of stakeholders engaged, and phase(s) of research in which engagement occurred, from identifying research questions to sharing study results [36]. Within article topic type, we searched the Framework, Editorial, Commentary category in the PCORI database because it includes “manuscripts that express a theoretical view on engagement in health research, including scientific commentaries, opinion briefs, or conceptual pieces such as models or frameworks” [36]. Furthermore, we completed a hand search of the supplemental data from the review by Jull et al. [7]. Given the volume of included studies, we did not conduct a grey literature search.

### Step 3: selecting the studies

Title and abstract screening included articles that (1) identified as a research partnership (minimum of one researcher associated with an academic institution and one partner such as an organization, clinician, patient, caregiver, policy-maker or decision-maker) [7, 10, 12], (2) referred to a TMF for the partnership, (3) were specific to health, (4) were published between January 2005 and June 2021, and (5) were written in English, the primary language of the research team. We excluded articles if they lacked an abstract or were a protocol paper, conference abstract, thesis, dissertation, commentary, opinion piece or editorial. During screening, we specifically looked for the “index” publication, namely a TMF’s first publication presenting its development as the definitive reference for the TMF [37]. However, not all TMFs were published in a way that it was possible to identify the first publication from the abstract. In these situations, if the article met the inclusion criteria, it was included in level 2 screening [37]. Prior to title/abstract screening, the first author (BT) pilot-tested the screening criteria on 50 articles and refined them to enhance clarity. Three teams of two reviewers completed title/abstract screening. All reviewers met prior to beginning screening to discuss the screening criteria. Each team completed a calibration exercise on 30 randomly selected articles to promote consistency in screening. Conflicts were resolved by consensus.

Full-text screening included index publications if they explicitly described (1) the TMF, (2) how the partner(s) were involved in the development of the TMF and (3) how the TMF informed the research partnership. We excluded the index publications if they were a book or commentary or they could not be retrieved with reasonable effort. Full-text screening occurred in two stages. First, we screened the full texts of index publications identified in title and abstract screening for inclusion. Secondly, we employed an ancestry and snowball search approach to locate the index publication from articles that referenced a TMF [29, 38]. This involved a hand search for the index publications via Google Scholar or our university library [29, 38]. There were no restrictions on when an index publication was published to be included in data analysis. Prior to full-text screening, the first author (BT) pilot-tested the screening criteria on 25 articles and refined them to improve clarity. One reviewer (BT) completed full-text screening. A calibration exercise was completed between three teams of two individuals on 12 randomly selected articles per team to ensure that the one reviewer was consistent in screening. The reviewer met every 2 weeks with the last author (KMS) to discuss concerns with full-text screening until it was completed. Both level 1 and 2 screening were completed on Rayyan (https://rayyan.qcri.org/welcome).

### Step4: data charting

An Excel data extraction form was developed a priori and pilot-tested by the first author on 10 randomly selected included articles. Through an iterative process, the data extraction form was revised to include information specific to (1) authors, (2) country of publication, (3) year of publication, (4) title of TMF, (5) intended users, (6) theoretical underpinning of TMF, (7) methodology, (8) methods utilized to develop the TMF, (9) purpose of the TMF, (10) extent of partner involvement in the development of the TMF as per the IAP2 Spectrum [18], (11) phase of research that the TMF related to [7], (12) concepts of knowledge user engagement identified by Jull et al. that the TMF related to [7], and (13) whether the TMF was graphically depicted by a figure or model. One reviewer (BT) completed data extraction on all included articles. A calibration exercise was conducted between two authors (BT and DS) on nine randomly selected articles to ensure the reviewer was accurate and consistent with data extraction. BT and KMS met virtually every 2 weeks to discuss data extraction until it was completed.

### Step 5: collating, summarizing and dissemination of results

Descriptive statistics were completed to identify the TMFs of health research partnerships including the number of index publications from which data were extracted. Additionally, we reported on counts and/or frequencies and proportions specific to the characteristics of the TMFs we extracted data on. A narrative synthesis was completed to describe the characteristics of the TMFs. A narrative synthesis is a systematic and transparent analysis approach utilized in reviews to examine and summarize text to explain the findings [39]. The research team employed an iterative process when collating and summarizing the findings to ensure consensus.

## Results

### Identifying TMF of health research partnerships

**Fig. 1:**
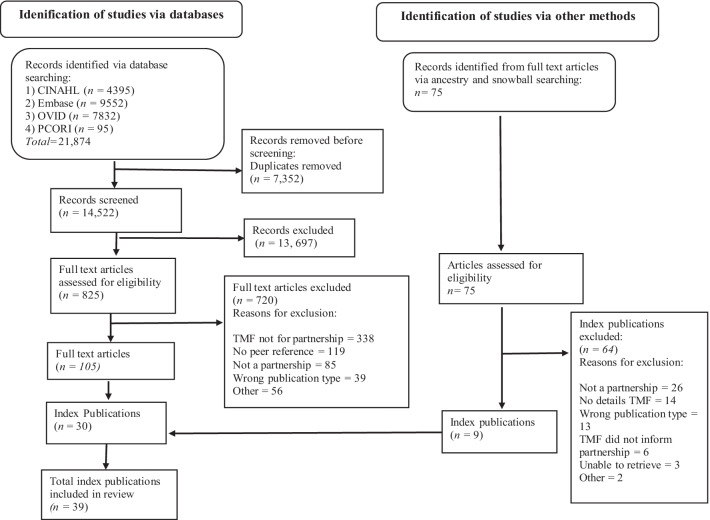
PRISMA flowchart [40]

See Fig. 1 for our PRISMA flowchart [40]. Thirty index publications were identified after full-text screening. We conducted an ancestry and snowball search for index publications on an additional 75 articles, which yielded another nine index publications. During the ancestry and snowball search we did not know which TMF was referenced in the article until we completed full-text screening. At times, the TMF we located from the ancestry and snowball search had already been identified in previous screening. Once screening was completed, 39 articles which described the development of a model or framework of health research partnerships were included for data analysis [41–79]. No articles describing theories were included. Moving forward we refer to models and frameworks (MFs) only.

### Characteristics of MF of health research partnerships

See Table 2 for characteristics of included articles. Twenty-four articles (62%) were published in the United States. Most articles (*n* = 30, 77%) did not explicitly indicate a methodology. When they did, qualitative methodology was the predominant methodology reported (*n* = 8, 21%). Only two articles (5%) indicated that the MFs developed were underpinned by theory.

**Table 2:** Characteristics of included model or framework (*n* = 39)

Study (country)	Year	Title	Intended users	Theoretical underpinning	Methodology	Figure or table
de Crespigny et al. [48] (Australia)	2004	Partnership model for ethical Indigenous research	Researchers partnering with Aboriginal groups	NR	NR	Figure
Bernier et al. [47] (Canada)	2006	NR	Between university chairs and partners	NR	NR	Table
Hewlett et al. [50] (United Kingdom)	2006	FIRST model	NR	NR	NR	Table
Anderson et al. [43] (United States)	2007	Partnership model	NR	NR	NR	Figure
McKay et al. [55] (United States)	2007	NR	NR	NR	NR	Figure
Silka et al. [58] (United States)	2008	Working Together model	Any type of research partnership	NR	NR	Figure
Wallerstein et al. [59] (United States)	2008	NR	NR	NR	NR	Figure
Jones et al. [52] (United States)	2009	Circle of Influence Model	NR	NR	NR	Figure
Warburton et al. [60] (Australia)	2009	NR	Researchers partnering with adults/older populations	NR	NR	NR
Abma and Broerse [41] (Netherlands)	2010	Dialogue model	NR	NR	NR	NR
James et al. [51] (United States)	2011	NR	NR	NR	Qualitative	Figure
Lindau et al. [53] (United States)	2011	NR	Large-scale health research partnerships	NR	NR	Both
Andrews et al. [44] (United States)	2012	CBPR Partnership Readiness Model	NR	NR	Qualitative	Figure
Baquet [45] (United States)	2012	NR	Partnerships between academic health centres and communities	Sociological framework, empowerment theory	NR	Figure
Sadler et al. [56] (United States)	2012	NR	Any type of research partnership	NR	NR	Table
Allen et al. [42] (United States)	2013	NR	NR	NR	NR	Table
Baquet et al. [46] (United States)	2013	NR	Partnerships between academic health centres and rural communities	NR	NR	Table
Deverka et al. [49] (United States)	2013	NR	NR	NR	NR	Figure
Martin del Campo et al. [54] (United States)	2013	BxCRRB model	NR	NR	NR	NR
Shippee et al. [57] (United States)	2013	NR	Patient and service user engagement research	NR	NR	Figure
CIHR [61] (Canada)	2014	Patient Engagement Framework	SPOR partners	NR	NR	Figure
Frank et al. [62] (United States)	2015	NR	Patient engagement research	NR	NR	Figure
King et al. [63] (United States)	2015	Community–academic partnership framework	NR	NR	Qualitative	Figure
Tse et al. [64] (United States)	2015	NR	NR	NR	Qualitative	Figure
Belone et al. [65] (United States)	2016	NR	NR	Socio-ecological framework	NR	Figure
McNeil et al. [67] (Canada)	2016	NR	Researchers partnering with older adults	NR	NR	Figure
Di Lorito et al. [68] (United Kingdom)	2017	NR	Researchers partnering with people with dementia	NR	NR	Table
Sheridan et al. [69] (United States)	2017	PCORI Engagement Rubric	Researchers apply for PCOR funding or any type of engaged research	NR	Qualitative	Figure
Corbie-Smith et al. [70] (United States)	2018	Engaged scholarship ethics framework	NR	NR	NR	Both
Dave et al. [71] (United States)	2018	NR	Community–academic partnerships	NR	Mixed Methods	Table
Gousse et al. [72] (United States)	2018	3Ps framework	Researchers partnering with Black, heterosexual men with HIV (or comparable group)	NR	NR	NR
Hamilton et al. [73] (Canada)	2018	PEIR framework	NR	NR	Qualitative	Table
Jull et al. [66] (Canada)	2018	NR	NR	NR	NR	Figure
Evans et al. [74] (United Kingdom)	2019	SUCCESS model	Researchers partnering with carers of and/or individuals with chronic conditions	NR	NR	Table
Key et al. [75] (United States)	2019	NR	NR	NR	NR	Figure
Swarbrick et al. [76] (United Kingdom)	2019	COINED Model	Researchers partnering with people with dementia	NR	NR	Figure
Di Lorito et al. [77] (United Kingdom)	2020	NR	Researchers partnering with carers of people with dementia or with members of the public	NR	Qualitative	Figure
Roche et al. [78] (Canada)	2020	Valuing All Voices Framework	Patient engagement research	NR	Qualitative	Both
Ward et al. [79] (Canada)	2020	NR	Non-Innu researchers partnering with Innu communities or any Indigenous community	NR	NR	Both

*BxCRRB* Bronx Community Research Review Board, *CIHR* Canadian Institutes of Health Research, *COINED* CO-Researcher INvolvement and Engagement in Dementia, *FIRST* facilitate, identify, respect, support and train, *NR* not reported, *PCOR* patient-centred outcomes research, *PEIR* patient engagement in research, *SPOR* Strategy for Patient-Oriented Research, *SUCCESS* Service Users with Chronic Conditions Encouraging Sensible Solutions

Table 3 depicts the methods explicitly reported to develop the MFs. Literature review (*n* = 11, 28%) and meetings (*n* = 10, 26%) were the predominant methods utilized, whereas systematic review (*n* = 1, 3%) was the least used. The number of methods utilized to develop a single MF ranged from *n* = 1 to *n* = 4. Eight articles (21%) did not report the methods utilized to develop the MFs.

**Table 3:** Methods utilized to develop model or framework (*n* = 39)

Authors	Literature review	Systematic review	Interviews	Focus group	Concept mapping	Workshop	Meetings	Survey	Other
de Crespigny et al. [48]	●		●	●				●	
Bernier et al. [47]	●						●		
Hewlett et al. [50]							●	●	Conferences
Anderson [43]									
Mckay et al. [55]									
Silka et al. [58]							●		Needs assessment
Wallerstein et al. [59]								●	
Jones et al. [52]									
Warburton et al. [60]						●			
Abma and Broerse [41]	●		●						Case studies
James et al. [51]			●				●		
Lindau et al. [53]							●		
Andrews et al. [44]			●	●					
Baquet [45]									
Sadler et al. [56]	●						●		
Allen et al. [42]							●		
Baquet et al. [46]	●						●		Strategic planning process
Deverka et al. [49]	●								Practical experience from a partnership
Martin del Campo et al. [54]	●			●					Conference calls, site visit
Shippee et al. [57]		●							Environmental scan, manual search of literature
CIHR [61]						●			
Frank et al. [62]	●								
King et al. [63]			●	●					
Tse et al. [64]				●			●		
Belone et al. [65]				●					
McNeil et al. [67]	●		●			●			Grey literature search, realist synthesis
Di Lorito et al. [68]	●								
Sheridan et al. [69]				●					Review of applications to PCOR to identify exemplar practices to guide development of rubric
Corbie-Smith et al. [70]	●		●			●		●	
Dave et al. [71]					●				
Gousse et al. [72]									
Hamilton et al. [73]									
Jull et al. [66]					●				
Evans et al. [74]				●		●	●		Normative group technique, email discussions
Key et al. [75]									Observations of community and academic partners, community dialogue sessions
Swarbrick et al. [76]							●	●	
Di Lorito et al. [77]									Personal reflections
Roche et al. [78]			●	●					
Ward et al. [79]									
Total	11	1	8	9	2	5	10	3	

The most frequently reported purpose of the MFs was to guide or manage (*n* = 14, 36%) a health research partnership. Sustaining the partnership was the least often reported purpose (*n* = 3, 8%). For more details on the purpose of the MFs, see Table 4.

**Table 4:** Purpose of model or framework (*n* = 39)

Authors	Plan	Guide/manage	Implement/conduct	Sustain	Support/enhance	Evaluate	Reflection (self and/or collective)	Policy and practice development	Other
de Crespigny et al. [48]									Enhance the reliability and validity of Indigenous research
Bernier et al. [47]		●							
Hewlett et al. [50]									Practical model for collaboration
Anderson et al. [43]		●							
McKay et al. [55]									Conceptual model of board development
Silka et al. [58]		●			●				
Wallerstein et al. [59]						●	●	●	Strengthen the CBPR research agenda on pathways and on relationships that may link CBPR processes and practices to CBPR system and capacity changes and health outcomes, inform research about partnership processes in CBPR epidemiologic or other assessment studies
Jones et al. [52]									To engage community and academic partners equally in an initiative to benefit the community while contributing to science
Warburton et al. [60]		●					●	●	To facilitate good-quality, multidisciplinary research
Abma and Broerse [41]									To complete agenda-setting in partnership research
James et al. [51]	●								
Lindau et al. [53]									Customizable framework for community engagement
Andrews et al. [44]									Indicate partnership readiness
Baquet [45]									Community and academic engagement in research
Sadler et al. [56]		●	●						Orient and provide a framework for research partners (community and university), train future academic and community members in collaborative health research
Allen et al. [42]						●			
Baquet et al. [46]				●					
Deverka et al. [49]			●						Prioritize and design partnered CER
Martin del Campo et al. [54]									Community consultation on research projects
Shippee et al. [57]					●	●			Understanding and reporting PSUE, a standard structure and language for reporting and indexing
CIHR [61]									Establish key concepts, principles and areas for patient engagement to be adopted by all SPOR partners
Frank et al. [62]						●	●	●	Identify required elements for PCOR, provide a way to describe patient-centredness in research
King et al. [63]				●					Forming a community–academic partnership in a low-income community
Tse et al. [64]		●							
Belone et al. [65]					●	●	●	●	
McNeil et al. [67]	●	●							
Di Lorito et al. [68]									Good practice for peer research
Sheridan et al. [69]		●	●		●				Disseminate engaged research, evaluate applications for research funding, develop PCOR training materials, monitor research teams
Corbie-Smith et al. [70]							●	●	Ethical review and conduct of engaged scholarship
Dave et al. [71]		●	●			●	●	●	
Gousse et al. [72]	●	●	●						
Hamilton et al. [73]	●	●	●			●			
Jull et al. [66]		●							Lay out steps and create opportunities for community–research collaboration
Evans et al. [74]									Involve public members in research
Key et al. [75]	●	●			●				Researchers can use to identify their level of engagement
Swarbrick et al. [76]									How to involve people with dementia in research
Di Lorito et al. [77]									Model for good practice in research
Roche et al. [78]		●	●					●	
Ward et al. [79]			●	●					Open and build relational spaces
Total	5	14	8	3	5	7	6	7	

*CER* comparative effectiveness research, *CIHR* Canadian Institutes of Health Research, *PCOR* patient-centred outcomes research, *PSUE* patient and service user engagement, *SPOR* Strategy for Patient-Oriented Research

**Fig. 2:**
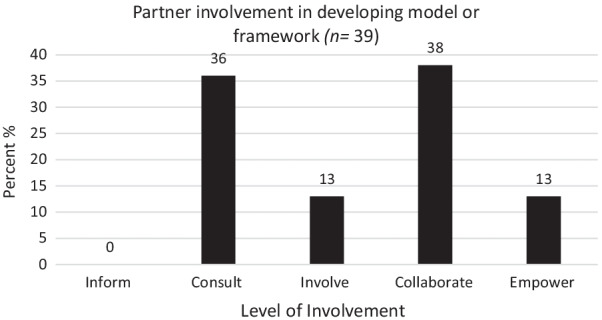
Level of knowledge user involvement in developing model or framework (*n* = 39) based on the IAP2 [18]

Figure 2 highlights the level of partner involvement in developing the MFs. Most MFs (*n* = 15, 38%) were developed using collaboration. For details specific to the phase of the research process the MF could be applied to, that is prepare, plan, conduct or apply, see Additional file 3.

### Concepts of knowledge user engagement

Specific to the 15 concepts of knowledge user engagement, we found that ethics—principles/values (*n* = 36, 92%) was the concept most often represented in the identified MFs (Table 5). Relational process (*n* = 31, 79%), knowledge user—prepare, support (*n* = 26, 67%) and resources (*n* = 26, 67%) were also commonly represented. Methodology (*n* = 1, 3%) was the least represented concept. The number of concepts represented in each MF ranged from *n* = 3 to *n* = 12. The median of the total number of concepts represented across the 39 MFs was *n* = 7.

**Table 5:** MFs (*n* = *39*) mapped to Jull et al.’s 15 concepts of knowledge user engagement [7]

Authors	Researcher: prepare, support	Knowledge user: prepare, support	Relational process	Research Agenda	Ethics: principles/values	Research Questions	Resources	Ethics: policy/rules	Methodology	Methods	Collect data	Analysis	Disseminate	Evaluate	Sustain	Total/15
de Crespigny et al. [48]			●	●	●											3
Bernier et al. [47]		●	●	●	●		●					●	●			7
Hewlett et al. [50]	●	●	●	●	●		●	●		●			●			9
Anderson et al. [43]			●	●	●											3
McKay et al. [55]	●	●	●		●		●	●		●	●	●	●		●	11
Silka et al. [58]	●	●		●	●	●	●			●	●	●	●		●	11
Wallerstein et al. [59]			●		●		●	●		●	●	●	●			8
Jones et al. [52]				●	●								●		●	4
Warburton et al. [60]		●		●	●	●			●	●		●	●			8
Abma and Broerse [41]	●	●	●	●	●											5
James et al. [51]			●	●	●										●	4
Lindau et al. [53]		●	●		●		●						●		●	6
Andrews et al. [44]	●	●	●		●		●	●							●	7
Baquet [45]	●	●	●		●		●						●	●	●	8
Sadler et al. [56]	●	●		●	●		●	●					●			7
Allen et al. [42]		●	●	●	●								●			4
Baquet et al. [46]	●	●	●	●	●	●	●	●			●		●	●		11
Deverka et al. [49]				●	●					●						3
Martin del Campo et al. [54]		●					●	●								3
Shippee et al. [57]	●	●	●	●		●	●			●	●	●	●	●		11
CIHR [61]	●	●	●	●	●	●	●	●		●	●	●				11
Frank et al. [62]	●	●	●	●	●		●	●		●	●	●	●			11
King et al. [63]			●		●		●	●								4
Tse et al. [64]	●		●		●											3
Belone et al. [65]			●		●		●	●		●			●			6
McNeil et al. [67]	●	●	●	●		●	●						●	●		8
Di Lorito et al. [68]	●	●	●		●		●									5
Sheridan et al. [69]	●	●	●		●	●	●			●	●	●	●	●		11
Corbie-Smith et al. [70]	●	●	●		●	●	●	●					●		●	9
Dave et al. [71]	●	●	●		●		●	●		●	●	●	●			10
Gousse et al. [72]	●	●			●		●						●		●	6
Hamilton et al. [73]		●	●		●		●									4
Jull et al. [66]				●	●			●								3
Evans et al. [74]			●		●		●									3
Key et al. [75]			●	●	●								●			4
Swarbrick et al. [76]	●	●	●	●	●		●	●		●	●	●	●	●		12
Di Lorito et al. [77]		●	●		●		●				●	●	●			7
Roche et al. [78]	●	●	●		●			●								5
Ward et al. [79]	●		●		●			●								4
Total/39	21	26	31	19	36	8	26	17	1	13	11	12	23	6	9	

*CIHR* Canadian Institutes of Health Research

## Discussion

We conducted a scoping review which identified and described 39 MFs of health research partnerships, but we did not identify any theory. Theory is utilized to predict and explain aspects of phenomena such as the success or failure of health research partnerships [24, 25, 80]. We did not aim to examine the success or failure of health research partnerships, or to identify factors that predict successful partnerships, and this may explain why we did not identify any theory. Furthermore, unlike theory, MFs are organizational templates that may be utilized to guide a health research partnership [26]. Our scoping review sought to identify the TMFs that were utilized to inform aspects of the health research partnership, that is, to guide the steps necessary for a health research partnership, which may also account for why we only identified MFs being used.

All MFs had representation from at least three concepts of knowledge user engagement, and no MFs encompassed all 15 concepts. We found that ethics—principles/values was the most represented concept in the MFs identified in our scoping review (Table 5). Jull et al. [7] described ethics—principles/values as “conduct knowledge user-researcher partnership work in an ethical way demonstrated by reflection on ethical concepts, and/or concern with particular values and research conducted in ways reported as meaningful, respectful, inclusive of those in the research partnership” (p. 7) (Table 1). Our scoping review sought to identify the TMFs which explicitly included concepts which influenced the research partnership, and this might explain why ethics—principles/values was most represented in our study. Relevancy, respect and inclusivity have all been identified as facilitators of health research partnerships [21, 81]. Partners embarking on a collaborative research project and developing an MF to inform the partnership may include aspects of relevancy, respect and inclusivity in the MF knowing they are facilitators of partnerships. Therefore, it might not be unexpected that we found explicit descriptions of ethics—principles/values in nearly all the MFs we identified in our study. We feel this is an encouraging finding, as it suggests that researchers and knowledge users collaborating in health research partnerships position ethical considerations as an important concept underlying their partnerships. While not examined in our scoping review, we speculate that health research partnerships underpinned by ethical principles and values may influence the success of these partnerships and would be a valuable topic for future research.

Like Jull et al. [7], we found variability in the number of concepts of knowledge user engagement represented within the included MFs. Specific to our study, the concepts ranged from 3 to 12 (Table 5). One explanation for this variability may be related to our full-text screening criteria. We included MFs that consisted of concepts to inform aspects of the health research partnership. However, several of the identified MFs also included additional concepts of knowledge user engagement, namely in dissemination, sustainability or evaluation. We did not exclude MFs if they captured these other aspects of knowledge user engagement. For instance, Swarbrick et al. [76] developed the COINED (CO-Researcher INvolvement and Engagement in Dementia) model, and we found that it had the largest number of concepts of knowledge user engagement represented in it (*n* = 12) (see Table 5) [76]. The COINED model not only included concepts that were partnership-focused (i.e. researcher—prepare, support; knowledge user—prepare, support; relational processes; and ethics—principles/values), but it also included concepts specific to the research process (i.e. research agenda, methods, data collection, analysis, dissemination and evaluation) [76]. Therefore, the COINED model had the largest number of knowledge user concepts represented in it [76]. In contrast, one of the frameworks with the fewest concepts was that of Ward et al. [79]. We mapped four knowledge user concepts represented in the framework: researcher—prepare, support; relational process; ethics—principles/values; and ethics—policy/rules (Table 5) [79]. These four concepts are underpinned by ideas such as power-sharing, trust, respect, inclusivity and developing meaningful research for all partners, which reflect a focus on the partnership as opposed to the research process itself [7]. Because this framework by Ward et al. [79] was focused on relational aspects of the partnership, it only included four concepts of knowledge user engagement and did not include concepts reflective of other aspects of the research process such as methods, data analysis, dissemination or evaluation.

Regardless of the number of concepts of knowledge user engagement identified within each MF, we cannot infer the quality or usability of the MF. Without a quality appraisal of the MFs, we cannot state that one MF is better than another. Instead, we suggest that future research could utilize an established evaluation tool, such as the Centre of Excellence for Partnership with Patients and the Public (CEPPP) evaluation tool, to assess the MFs for scientific rigour, involvement of knowledge users in their development, and their usability [82]. The CEPPP has been utilized in previously published research which evaluated the quality of frameworks for patients and the public involved in research [29]. A quality appraisal of the MFs could provide researchers and knowledge users with information to help them choose an MF appropriate for their health research partnership. Additionally, a quality appraisal of MFs may encourage their utilization, thus facilitating partnerships between researchers and knowledge users.

As one of our objectives was to map the concepts of knowledge user engagement to the identified MFs, we decided that we would extract these concepts only if they were explicitly represented in an MF—that is, the concept of knowledge user engagement had to be clearly represented in either a graphical depiction of the MF or described in the text of the article. We opted for this coding approach to maintain objectivity and provide researchers and knowledge users interested in MFs of health research partnerships with an accurate depiction of the concepts of knowledge user engagement within each MF we identified. As we read an MF, we referred to the descriptions of the concepts provided by Jull et al. [7] and utilized the descriptions to determine whether the concept of knowledge user engagement was explicitly mentioned. For instance, Jull et al. [7] described the concept of methodology as follows: “[d]ecide on the research methodology (approach) or report process to justify the use of the proposed methodology” (p. 7). When we searched for representation of methodology in an MF, we read the text and/or reviewed the graphical depiction specifically looking for the terms “methodology” or “approach” or “report on process”. If we did not find these terms within the MF, we coded the concept as not represented. We acknowledge that this was a strict approach to employ. We believe it may explain why some of the MFs we identified included a smaller number of concepts of knowledge user engagement than other MFs. However, we believe our results mapping the concepts of knowledge user engagement to the MFs are helpful for researchers and knowledge users embarking on a collaborative research project. They can refer to our results for an MF to plan, guide, implement, enhance or sustain the partnership and review the concepts of knowledge user engagement represented in the MFs to determine which MF may meet their needs. The researchers and knowledge users can then seek out the MF for further information about it.

## Strengths and limitations

Strengths of our scoping review included our use of the methodological frameworks by Arksey and O’Malley [30] and Levac et al. [31] to guide the systematic approach we undertook to promote rigour for our scoping review. Specifically, we liaised with a research librarian to develop the research question and search strategy which included a relevant time frame, key search terms and multiple databases to ensure we captured the most appropriate articles for inclusion. Additionally, we utilized the PRISMA-ScR to provide guidance on reporting our scoping review [32].

One limitation of our study was deviation from our scoping review protocol. We had planned for two independent reviewers during full-text screening and data extraction to enhance methodological rigour, but title and abstract screening took longer than anticipated due to the high volume of articles included. Reviewers were no longer available to assist with full-text screening and data extraction because they were required for other projects. To maintain rigour, we completed pilot testing and multiple calibration exercises of our full-text screening criteria and data extraction form. Additionally, BT and KMS met every 2 weeks during data extraction to discuss the extraction process. Despite not having two independent reviewers for full-text and data extraction, we are confident our processes for full-text screening and data extraction maintained rigour. A further limitation of our study was the exclusion of non-English articles and articles with no abstracts.

We acknowledge that we did not involve knowledge users in our study. Now that we have identified and described MFs of health research partnerships, we feel it is necessary to better understand knowledge users’ perspectives of MFs that inform the partnership process. Future research could explore knowledge users’ attitudes, beliefs and experiences specific to MFs of the health research partnerships.

## Conclusion

Our study aimed to identify and describe the characteristics of TMFs of health research partnerships, and to map concepts of knowledge user engagement to the TMFs. We identified 39 models or frameworks of health research partnerships, which we defined as a partnership between an academically affiliated researcher(s) and non-academically affiliated partner(s). Of significance, no theory of health research partnerships was identified, which may limit the ability to explain or predict successful health research partnerships. Encouragingly, the concept of ethical principles and values was one of the most frequently represented in the MFs. This suggests that ethical considerations are an important concept informing partnerships between researchers and knowledge users and may enhance successful health research partnerships. We believe our findings are valuable to researchers and knowledge users partnering on a research project. The models or frameworks we identified could be sought out by partners and utilized to inform aspects of the health research partnership process, such as guiding or managing a partnership. Ultimately, this may contribute to research that is more relevant to the knowledge users, thus enhancing the utilization of evidence in healthcare practice and improving health outcomes and the efficiency of a healthcare system or organization.

## Supplementary Information

**Additional file 1.** Descriptions of level of public involvement as per International Association for Public Participation [18].

**Additional file 2.** Search strategy.

**Additional file 3.** Phase of research process that model or framework aligns with.

## Data Availability

The datasets used and/or analysed during the current study are available from the corresponding author on request.

## Abbreviations

BxCRRB: Bronx Community Research Review Board
CEPPP: Centre of Excellence for Partnership with Patients and the Public
CBPR: Community-based participatory research
CIHR: Canadian Institutes of Health Research
COINED: CO-Researcher INvolvement and Engagement in Dementia
CER: Comparative effectiveness research
FIRST: Facilitate, identify, respect, support and train
IAP2: International Association for Public Participation
MFs: Models and frameworks
PCOR: Patient-centred outcomes research
PCORI: Patient-Centred Outcomes Research Institute
PEIR: Patient engagement in research
PSUE: Patient and service user engagement
SPOR: Strategy for Patient-Oriented Research
SUCCESS: Service Users with Chronic Conditions Encouraging Sensible Solutions
TMFs: Theories, models, and frameworks

## References

[1] Davis D, Taylor-Vaisey A (1997). A systematic review of theoretic concepts, practical experience and research evidence in the adoption of clinical practice guidelines. Can Med Assoc J.

[2] Grol R (2001). Successes and failures in the implementation of evidence-based guidelines for clinical practice. Med Care.

[3] Grimshaw J, Eccles M, Lavis J, Hill S, Squires J (2012). Knowledge translation of research findings. Implement Sci.

[4] Chassin M, Galvin R (1998). The urgent need to improve health care quality. JAMA.

[5] Krzyzanowska MK, Kaplan R, Sullivan R (2011). How may clinical research improve healthcare outcomes?. Ann Oncol.

[6] Bowen S, Graham I, Straus SETJ, Graham ID (2013). Integrated knowledge translation. Knowledge translation in healthcare.

[7] Jull JE, Davidson L, Dungan R, Nguyen T, Woodward KP, Graham ID (2019). A review and synthesis of frameworks for engagement in health research to identify concepts of knowledge user engagement. BMC Med Res Methodol.

[8] Bowen S, Botting I, Graham ID (2019). Experience of health leadership in partnering with university-based researchers in Canada—a call to "re-imagine" research. Int J Health Policy Manag.

[9] Graham ID, Kothari A, McCutcheon C, Integrated Knowledge Translation Research Network Project (Leads) (2018). Moving knowledge into action for more effective practice, programmes and policy: protocol for a research programme on integrated knowledge translation. Implement Sci.

[10] Hoekstra F, Mrklas KJ, Sibley KM (2018). A review protocol on research partnerships: a Coordinated Multicenter Team approach. Syst Rev.

[11] Nguyen T, Graham ID, Mrklas KJ (2020). How does integrated knowledge translation (IKT) compare to other collaborative research approaches to generating and translating knowledge? Learning from experts in the field. Health Res Policy Syst.

[12] Drahota A, Meza R, Brikho B (2016). Community-academic partnerships: a systematic review of the state of the literature and recommendations for future research. Milbank Q.

[13] Gagliardi AR, Berta W, Kothari A, Boyko J, Urquhart R (2016). Integrated knowledge translation (IKT) in health care: a scoping review. Implement Sci.

[14] Binet A, Gavin V, Carroll L, Arcaya M (2019). Designing and facilitating collaborative research design and data analysis workshops: lessons learned in the healthy neighborhoods study. Int J Environ Res Public Health.

[15] Roberge-Dao J, Yardley B, Menon A (2019). A mixed-methods approach to understanding partnership experiences and outcomes of projects from an integrated knowledge translation funding model in rehabilitation. BMC Health Serv Res.

[16] Swartz LJ, Callahan KA, Butz AM (2004). Methods and issues in conducting a community-based environmental randomized trial. Environ Res.

[17] Hoekstra F, Mrklas KJ, Khan M (2020). A review of reviews on principles, strategies, outcomes and impacts of research partnerships approaches: a first step in synthesising the research partnership literature. Health Res Policy Syst.

[18] IAP2. IAP2 Spectrum. International Association for Public Participation. 2021. https://iap2canada.ca/Resources/Documents/0702-Foundations-Spectrum-MW-rev2%20(1).pdf. Accessed 20 July 2021.

[19] McNeill M, Noyek S, Engeda E, Fayed N (2021). Assessing the engagement of children and families in selecting patient-reported outcomes (PROs) and developing their measures: a systematic review. Qual Life Res.

[20] Jull J, Giles A, Graham ID (2017). Community-based participatory research and integrated knowledge translation: advancing the co-creation of knowledge. Implement Sci.

[21] Zych MM, Berta WB, Gagliardi AR (2019). Initiation is recognized as a fundamental early phase of integrated knowledge translation (IKT): qualitative interviews with researchers and research users in IKT partnerships. BMC Health Serv Res.

[22] Boland L, Kothari A, McCutcheon C, Graham ID, Integrated Knowledge Translation Research Network (2020). Building an integrated knowledge translation (IKT) evidence base: colloquium proceedings and research direction. Health Res Policy Syst.

[23] Graham ID, Tetroe J (2007). Some theoretical underpinnings of knowledge translation. Acad Emerg Med.

[24] Kerlinger F (1986). Foundations of behavioral research.

[25] Walker L, Avant K (2019). Strategies for theory construction in nursing.

[26] Nilsen P (2015). Making sense of implementation theories, models and frameworks. Implement Sci.

[27] Bergstrom A, Ehrenberg A, Eldh AC (2020). The use of the PARIHS framework in implementation research and practice-a citation analysis of the literature. Implement Sci.

[28] Zych MM, Berta WB, Gagliardi AR (2020). Conceptualising the initiation of researcher and research user partnerships: a meta-narrative review. Health Res Policy Syst.

[29] Greenhalgh T, Hinton L, Finlay T (2019). Frameworks for supporting patient and public involvement in research: systematic review and co-design pilot. Health Expect.

[30] Arksey H, O'Malley L (2005). Scoping studies: towards a methodological framework. Int J Soc Res Methodol.

[31] Levac D, Colquhoun H, O’Brien K (2010). Scoping studies: advancing the methodology. Implement Sci.

[32] Tricco AC, Lillie E, Zarin W (2018). PRISMA extension for scoping reviews (PRISMA-ScR): checklist and explanation. Ann Intern Med.

[33] Tittlemier BJ SK. A scoping review to identify and describe characteristics of theories, models and frameworks of health research partnerships. 2020. https://osf.io/qntym. Accessed 6th Apr 2021. 10.1186/s12961-022-00877-4 PMC9206347 35717196

[34] Esmail R, Hanson HM, Holroyd-Leduc J (2020). A scoping review of full-spectrum knowledge translation theories, models, and frameworks. Implement Sci.

[35] Strifler L, Cardoso R, McGowan J (2018). Scoping review identifies significant number of knowledge translation theories, models, and frameworks with limited use. J Clin Epidemiol.

[36] Engagement in Health Research Literature Explorer. Patient-Centred Outcomes Research Institute. 2021. https://www.pcori.org/engagement/engagement-literature. Accessed 17 May 2020.

[37] Sibley KM, Beauchamp MK, Van Ooteghem K, Straus SE, Jaglal SB (2015). Using the systems framework for postural control to analyze the components of balance evaluated in standardized balance measures: a scoping review. Arch Phys Med Rehabil.

[38] Greenhalgh T, Peacock R (2005). Effectiveness and efficiency of search methods in systematic reviews of complex evidence: audit of primary sources. BMJ.

[39] Popay J, Roberts H, Sowden A (2006). Narrative synthesis in systematic reviews: a product from the ESRC methods programme. ESRC Methods Program.

[40] Page MJ, McKenzie JE, Bossuyt PM (2021). The PRISMA 2020 statement: an updated guideline for reporting systematic reviews. BMJ.

[41] Abma TA, Broerse JE (2010). Patient participation as dialogue: setting research agendas. Health Expect.

[42] Allen ML, Svetaz AV, Hurtado GA, Linares R, Garcia-Huidobro D, Hurtado M (2013). The developmental stages of a community-university partnership: the experience of Padres Informados/Jovenes Preparados. Prog Community Health Partnersh.

[43] Anderson NL, Calvillo ER, Fongwa MN (2007). Community-based approaches to strengthen cultural competency in nursing education and practice. J Transcult Nurs.

[44] Andrews JO, Newman SD, Meadows O, Cox MJ, Bunting S (2012). Partnership readiness for community-based participatory research. Health Educ Res.

[45] Baquet CR (2012). A model for bidirectional community-academic engagement (CAE): overview of partnered research, capacity enhancement, systems transformation, and public trust in research. J Health Care Poor Underserved.

[46] Baquet CR, Bromwell JL, Hall MB, Frego JF (2013). Rural community-academic partnership model for community engagement and partnered research. Prog Community Health Partnersh.

[47] Bernier J, Rock M, Roy M, Bujold R, Potvin L (2006). Structuring an inter-sector research partnership: a negotiated zone. Soz Praventivmed.

[48] de Crespigny C, Emden C, Kowanko I, Murray H (2004). A ‘partnership model’ for ethical Indigenous research. Collegian.

[49] Deverka PA, Lavallee DC, Desai PJ (2012). Stakeholder participation in comparative effectiveness research: defining a framework for effective engagement. J Comp Eff Res.

[50] Hewlett S, Wit M, Richards P (2006). Patients and professionals as research partners: challenges, practicalities, and benefits. Arthritis Rheum.

[51] James S, Guedy A, Bickell N (2011). Community ACTION boards: an innovative model for effective community-academic research partnerships. Prog Community Health Partnersh.

[52] Jones L, Wells K, Norris K, Meade B, Koegel P (2009). Chapter 1. The vision, valley, and victory of community engagement. Ethn Dis.

[53] Lindau ST, Makelarski JA, Chin MH (2011). Building community-engaged health research and discovery infrastructure on the South Side of Chicago: science in service to community priorities. Prev Med.

[54] Martin del Campo F, Casado J, Spencer P, Strelnick H (2013). The development of the Bronx Community Research Review Board: a pilot feasibility project for a model of community consultation. Prog Community Health Partnersh.

[55] McKay MM, Hibbert R, Lawrence R (2007). Creating mechanisms for meaningful collaboration between members of urban communities and university-based HIV prevention researchers. Soc Work Ment Health.

[56] Sadler LS, Larson J, Bouregy S (2012). Community-university partnerships in community-based research. Prog Community Health Partnersh.

[57] Shippee ND, Domecq Garces JP, Prutsky Lopez GJ (2015). Patient and service user engagement in research: a systematic review and synthesized framework. Health Expect.

[58] Silka L, Cleghorn GD, Grullon M, Tellez T (2008). Creating community-based participatory research in a diverse community: a case study. J Empir Res Hum Res Ethics.

[59] Wallerstein N, Oetzel J, Duran B, Tafoya G, Belone L, Rae R, Minkler M, Wallerstein N (2008). What predicts outcomes in CBPR?. Community-based participatory research for health: from process to outcomes.

[60] Warburton J, Bartlett H, Carroll M, Kendig H (2009). Involving older people in community-based research: Developing a guiding framework for researchers and community organisations. Australas J Ageing.

[61] CIHR. Patient engagement framework. Ottawa, Canada. 2014.

[62] Frank L, Forsythe L, Ellis L (2015). Conceptual and practical foundations of patient engagement in research at the patient-centered outcomes research institute. Qual Life Res.

[63] King K, Morris D, Jones L (2015). The Los Angeles healthy community neighborhood initiative: a ten year experience in building and sustaining a successful community-academic partnership. HSOA J Community Med Public Health Care.

[64] Tse AM, Palakiko DM, Daniggelis E, Makahi E (2015). Facilitating community participants' research engagement: community members' perceptions of community-based research. Int J Nurs Clin Pract.

[65] Belone L, Lucero JE, Duran B (2016). Community-based participatory research conceptual model: community partner consultation and face validity. Qual Health Res.

[66] Jull J, Giles A, Boyer Y, Stacey D, Lodge M (2018). Development of a collaborative research framework: an example of a study conducted by and with a First Nations, Inuit and Métis Women’s community and its research partners. ACME Int J Crit Geogr.

[67] McNeil H, Elliott J, Huson K (2016). Engaging older adults in healthcare research and planning: a realist synthesis. Res Involv Engagem.

[68] Di Lorito C, Birt L, Poland F (2017). A synthesis of the evidence on peer research with potentially vulnerable adults: how this relates to dementia. Int J Geriatr Psychiatry.

[69] Sheridan S, Schrandt S, Forsythe L, Hilliard TS, Paez KA, Advisory Panel on Patient Engagement (2017). The PCORI engagement rubric: promising practices for partnering in research. Ann Fam Med.

[70] Corbie-Smith G, Wynn M, Richmond A (2018). Stakeholder-driven, consensus development methods to design an ethical framework and guidelines for engaged research. PLoS ONE.

[71] Dave G, Frerichs L, Jones J (2018). Conceptualizing trust in community-academic research partnerships using concept mapping approach: a multi-CTSA study. Eval Progr Plann.

[72] Gousse Y, McFarlane D, Fraser M (2018). Lessons learned from the implementation of a shared community-academic HIV prevention intervention. Prog Community Health Partnersh.

[73] Hamilton CB, Hoens AM, Backman CL (2018). An empirically based conceptual framework for fostering meaningful patient engagement in research. Health Expect.

[74] Evans BA, Porter A, Snooks H, Burholt V (2019). A co-produced method to involve service users in research: the SUCCESS model. BMC Med Res Methodol.

[75] Key KD, Furr-Holden D, Lewis EY (2019). The continuum of community engagement in research: a roadmap for understanding and assessing progress. Prog Community Health Partnersh.

[76] Swarbrick CM, Doors O, Davis K, Keady J, Scottish Dementia Working Group, EDUCATE (2019). Visioning change: co-producing a model of involvement and engagement in research (Innovative Practice). Dementia (Lond).

[77] Di Lorito C, Godfrey M, Dunlop M (2020). Adding to the knowledge on patient and public involvement: reflections from an experience of co-research with carers of people with dementia. Health Expect.

[78] Roche P, Shimmin C, Hickes S (2020). Valuing All Voices: refining a trauma-informed, intersectional and critical reflexive framework for patient engagement in health research using a qualitative descriptive approach. Res Involv Engagem.

[79] Ward LM, Hill MJ, Chreim S, Poker C, Olsen Harper A, Wells S (2020). Developing an Innu framework for health research: the canoe trip as a metaphor for a collaborative approach centered on valuing Indigenous knowledges. Soc Sci Med.

[80] Glanz K, Bishop DB (2010). The role of behavioral science theory in development and implementation of public health interventions. Annu Rev Public Health.

[81] Beckett K, Farr M, Kothari A, Wye L, le May A (2018). Embracing complexity and uncertainty to create impact: exploring the processes and transformative potential of co-produced research through development of a social impact model. Health Res Policy Syst.

[82] CEPPP. A Scorecard for Evaluating Engagement. The Center of Excellence for Partnership with Patients and the Public. 2021. https://ceppp.ca/en/evaluation-toolkit/a-scorecard-for-evaluating-engagement/. Accessed 26th Aug 2021.

