# Supplementary material for: A scoping review to identify and describe the characteristics of theories, models and frameworks of health research partnerships
Source: Health Res Policy Syst. 2022 Jun 18;20:69. doi: 10.1186/s12961-022-00877-4 (PMC9206347; doi:10.1186/s12961-022-00877-4)
Supplement: Supplementary file 1 — Additional file 1. Descriptions of level of public involvement as per International Association for Public Participation [18]. [file 12961_2022_877_MOESM1_ESM.docx]

Additional File 1. Descriptions of level of public involvement as per International Association of Public Participation^18^

| Inform | Consult | Involve | Collaborate | Empower |
| --- | --- | --- | --- | --- |
| **Provide public with balanced and objective information to assist them in understanding the problem, alternatives and/or solutions** | Obtain public feedback on analysis, alternatives and/or decision | Work directly with the public throughout the process to ensure that public concerns and aspirations are consistently understood and considered | Partner with the public in each aspect of the decision including the development of alternatives and the preferred solution | Place final decision-making in the hands of the public. |
