# Supplementary material for: A scoping review to identify and describe the characteristics of theories, models and frameworks of health research partnerships
Source: Health Res Policy Syst. 2022 Jun 18;20:69. doi: 10.1186/s12961-022-00877-4 (PMC9206347; doi:10.1186/s12961-022-00877-4)
Supplement: Supplementary file 2 — Additional file 2. Search strategy. [file 12961_2022_877_MOESM2_ESM.docx]

Additional File 2. Search Strategy

Database: Ovid MEDLINE(R) ALL <1946 to May 19, 2020>

Search Strategy:

--------------------------------------------------------------------------------

1 models, educational/ or models, nursing/ or models, organizational/ or models, psychological/ (82990)

2 (model* adj3 (education* or instruction* or mental or psychologic* or nursing or organizational)).tw,kf. (15180)

3 framework*.tw,kf. (272854)

4 theor*.tw,kf. (647517)

5 model*.tw,kf. (2873297)

6 1 or 2 or 3 or 4 or 5 (3514078)

7 Community-Based Participatory Research/ (4311)

8 ((community or consumer) adj3 "based research").tw,kf. (896)

9 "CBPR".tw,kf. (1134)

10 Community Participation/ (17052)

11 ((community or consumer) adj3 participation).tw,kf. (5633)

12 action research.tw,kf. (4195)

13 integrated knowledge translation.tw,kf. (203)

14 patient.mp. and public involvement.tw,kf. [mp=title, abstract, original title, name of substance word, subject heading word, floating sub-heading word, keyword heading word, organism supplementary concept word, protocol supplementary concept word, rare disease supplementary concept word, unique identifier, synonyms] (795)

15 patient.mp. and public engagement.tw,kf. [mp=title, abstract, original title, name of substance word, subject heading word, floating sub-heading word, keyword heading word, organism supplementary concept word, protocol supplementary concept word, rare disease supplementary concept word, unique identifier, synonyms] (201)

16 community-based research.tw,kf. (851)

17 community engaged research.tw,kf. (282)

18 community engagement.tw,kf. (2692)

19 engagement research.tw,kf. (64)

20 community-academic partnership*.tw,kf. (341)

21 community-academic research.tw,kf. (34)

22 patient collaboration.tw,kf. (125)

23 stakeholder collaboration.tw,kf. (114)

24 research partnership.tw,kf. (414)

25 partnered research.tw,kf. (78)

26 patient-oriented research.tw,kf. (287)

27 (mode two adj3 knowledge).tw,kf. (1)

28 (mode II adj3 knowledge).tw,kf. (2)

29 engaged scholarship.tw,kf. (57)

30 7 or 8 or 9 or 10 or 11 or 12 or 13 or 14 or 15 or 16 or 17 or 18 or 19 or 20 or 21 or 22 or 23 or 24 or 25 or 26 or 27 or 28 or 29 (33423)

31 6 and 30 (8571)

32 limit 31 to (english language and yr="2005 - 2020") (6592)

***************************
