# Supplementary material for: A scoping review to identify and describe the characteristics of theories, models and frameworks of health research partnerships
Source: Health Res Policy Syst. 2022 Jun 18;20:69. doi: 10.1186/s12961-022-00877-4 (PMC9206347; doi:10.1186/s12961-022-00877-4)
Supplement: Supplementary file 3 — Additional file 3. Phase of research process that model or framework aligns with. [file 12961_2022_877_MOESM3_ESM.docx]

Additional File 3. Phase of research process that model or framework aligns with

| Authors | Prepare | Plan | Conduct | Apply | Total |
| --- | --- | --- | --- | --- | --- |
| **de Crespigny et al**^48^ |  |  |  |  | 3 |
| **Bernier et al**^47^ |  |  |  |  | 4 |
| **Hewlett et al**^50^ |  |  |  |  | 3 |
| **Anderson et al**^43^ |  |  |  |  | 2 |
| **McKay et al**^55^ |  |  |  |  | 4 |
| **Silka et al**^58^ |  |  |  |  | 4 |
| **Wallerstein et al**^59^ |  |  |  |  | 4 |
| **Jones et al**^52^ |  |  |  |  | 3 |
| **Warburton et al**^60^ |  |  |  |  | 4 |
| **Abma and Broerse**^41^ |  |  |  |  | 2 |
| **James et al**^51^ |  |  |  |  | 3 |
| **Lindau et al**^53^ |  |  |  |  | 3 |
| **Andrews et al**^44^ |  |  |  |  | 4 |
| **Baquet**^45^ **(2012)** |  |  |  |  | 3 |
| **Sadler et al**^56^ |  |  |  |  | 3 |
| **Allen et al**^42^ |  |  |  |  | 3 |
| **Baquet et al**^46^ **(2013)** |  |  |  |  | 4 |
| **Deverka et al**^49^ |  |  |  |  | 2 |
| **Martin del Campo et al**^54^ |  |  |  |  | 2 |
| **Shippee et al**^57^ |  |  |  |  | 4 |
| **CIHR**^61^ |  |  |  |  | 3 |
| **Frank et al**^62^ |  |  |  |  | 4 |
| **King et al**^63^ |  |  |  |  | 2 |
| **Tse et al**^64^ |  |  |  |  | 2 |
| **Belone et al**^65^ |  |  |  |  | 4 |
| **Jull et al**^66^ |  |  |  |  | 2 |
| **McNeil et al**^67^ |  |  |  |  | 3 |
| **Di Lorito et al**^68^ **(2017)** |  |  |  |  | 2 |
| **Sheridan et al**^69^ |  |  |  |  | 4 |
| **Corbie-Smith et al**^70^ |  |  |  |  | 3 |
| **Dave et al**^71^ |  |  |  |  | 4 |
| **Gousse et al**^72^ |  |  |  |  | 3 |
| **Hamilton et al**^73^ |  |  |  |  | 2 |
| **Evans et al**^74^ |  |  |  |  | 2 |
| **Key et al**^75^ |  |  |  |  | 3 |
| **Swarbrick et al**^76^ |  |  |  |  | 4 |
| **Di Lorito et al**^77^ **(2020)** |  |  |  |  | 4 |
| **Roche et al**^78^ |  |  |  |  | 2 |
| **Ward et al**^79^ |  |  |  |  | 2 |
| Total | 39 | 39 | 14 | 26 |  |
